# Association Between Postpartum Depression and Personality Traits Among Japanese Postpartum Mothers and Fathers

**DOI:** 10.3390/jcm13247714

**Published:** 2024-12-18

**Authors:** Ayana Haku, Hitoshi Kaneko, Junko Kawahito

**Affiliations:** 1 Graduate School of Education and Human Development, Nagoya University, Nagoya 464-8601, Japan; 2 Psychological Support and Research Center for Human Development, Nagoya University, Nagoya 464-8601, Japan; 3 Department of Clinical Psychology, Graduate School of Medicine, Kagawa University, Takamatsu 761-0793, Japan; kawahito.junko@kagawa-u.ac.jp

**Keywords:** paternal postpartum depression, maternal postpartum depression, extraversion, neuroticism, marital relationship

## Abstract

**Background/Objectives**: Although numerous investigations have been conducted on postpartum depression, studies on the association between postpartum depression and personality traits of mothers and fathers are lacking. This study aimed to examine the association between postpartum depression and the Big Five personality models among Japanese mothers and fathers at one-month health check-ups. **Methods**: The participants were 82 couples, and they responded to the Edinburgh Postnatal Depression Scale (EPDS), the Japanese version of the Ten-Item Personality Inventory, and the Quality of Marriage Index (QMI). We examined the correlations among variables and analyzed the data using structural equation modeling (SEM). **Results**: Maternal neuroticism was significantly associated with maternal depression (β = 0.50, *p* < 0.001), and maternal extraversion was significantly associated with paternal depression (β = −0.64, *p* < 0.001). In addition, we found that maternal postpartum depression was associated with maternal marital satisfaction (r = −0.29, *p* = 0.037); however, this association disappeared in SEM. **Conclusions**: Our findings suggest that health practitioners should pay attention to depression and personality traits in both postpartum mothers and fathers. Moreover, we should consider the different associations between depression and personality in parents when initiating interventions.

## 1. Introduction

In Japan, problems related to the perinatal period are becoming more serious, and postpartum depression is one of the reasons. Postpartum depression is defined in the DSM-5 as a mood disorder that develops during pregnancy and within four weeks of childbirth [1]. Depressed mood, loss of interest, disturbed sleep and appetite, difficulty concentrating, psychomotor problems, and suicidal thoughts, among others are a common feature of postpartum depression and its clinical manifestation [2]. A meta-analysis of maternal postpartum depression in the Japanese population found that the prevalence of maternal postpartum depression was 15.1% within 1 month postpartum, 11.6% within 1–3 months, 11.5% within 3–6 months, and 11.5% within 6–12 months [3]. Rapid physical and lifestyle changes during the first month postpartum make it difficult for mothers to maintain their mental health. Maternal postpartum depression is associated with vulnerability to reproductive hormone fluctuations after childbirth [4]. In addition, previous studies showed that low education, unmarried status, unemployment, cesarean section, unplanned pregnancy, preterm delivery, and no breastfeeding are associated with the development of maternal postpartum depression [5]. Postpartum depression reduces maternal sensitivity and disrupts essential caregiving activities that establish sleep routines and ensure safety practices [6]. Systematic reviews have also suggested that postpartum depression contributes to the cognitive and socioemotional development of infants [7]. In addition, postpartum depression may lead to maternal suicide [8] and child abuse because it becomes difficult to bond with the child [9]. Adequate support for mothers with postpartum depression is important for the health of both parents and their children.

Fathers also suffer from mental illnesses after childbirth, and paternal postpartum depression has attracted attention in recent years [10]. Paternal postpartum depression is characterized by symptoms such as exhaustion, irritability, restlessness, and episodes of anger, which are reported to be more prominent than feelings of sadness [11]. A recent meta-analysis showed that the prevalence of paternal postpartum depression was 7.8% within one to three months and 8.8% within one year of childbirth [12]. Another meta-analysis reported that joblessness, low social support, adverse life events, perceived stress, financial pressure, psychiatric conditions, and mental illness were associated with the development of paternal postpartum depression [13]. In addition, paternal postpartum depression was shown to be associated with a high risk of suicide [14]. Furthermore, in Japan, paternal depression was significantly associated with child maltreatment tendencies [15]. Although diagnostic criteria for paternal postpartum depression have not yet been defined, adequate support for postpartum fathers is important for family health.

Several studies have reported that postpartum depression is associated with the Big Five personality traits, particularly neuroticism [16,17]. Previous studies used the Big Five personality models to assess personality traits among postpartum mothers. The Big Five has accumulated solid findings in recent personality trait theory [18]. The Big Five has a history that begins with Allport and Odbert’s [19] lexical approach and was established as a single personality theory by Goldberg [20]. This theory is based on an analysis of natural language terms used to describe ourselves and others, converging on five dimensions of basic personality traits [21]. The Big Five model describes personality in terms of five main trait dimensions: (a) neuroticism, (b) extraversion, (c) agreeableness, (d) conscientiousness, and (e) openness. In current personality research, the idea that five factors represent an individual’s personality traits is becoming the most common view. For example, Serra et al. [16] showed that neuroticism has a direct impact on maternal postpartum depression, and Kaźmierczak [17] indicated that the neuroticism of parents affects postpartum depression in partners. However, the findings of previous studies on postpartum fathers have been inconsistent. Shintya [22] reported that paternal extraversion and neuroticism were significantly associated with paternal postpartum depression, while Bielawska-Batorowicz and Kossakowska-Petrycka [23] indicated that paternal neuroticism may not be associated with paternal postpartum depression. Further research on paternal postpartum depression and personality traits is required to support postpartum fathers.

Research on the association between postpartum depression and the Big Five personality models is inconclusive and still lacking. Furthermore, despite the importance of understanding fathers’ and mothers’ mental health during the postpartum period [24], studies on postpartum depression using paired data are scarce.

Furthermore, marital satisfaction is considered an important factor that affects postpartum depression, as poor marital satisfaction has been reported to affect postpartum depression in mothers [25] and fathers [24]. A meta-analysis of 49 cases found that marital satisfaction undergoes a medium decrease during pregnancy through 12 months postpartum and decreases slightly from 12–24 months postpartum [26]. The decline in marital satisfaction for childless couples was less, and when marital satisfaction declined sharply, the other partner’s satisfaction also declined sharply [26].

This study examined the association between postpartum depression and the Big Five personality models among Japanese mothers and fathers during one-month postpartum check-ups. Marital satisfaction was also assessed. We hypothesized that partner postpartum depression would be positively associated with neuroticism [17] and paternal postpartum depression would be negatively associated with marital relationship [24].

## 2. Materials and Methods

### 2.1. Participants

The survey was distributed to 164 people who had undergone a one-month postpartum health checkup at Kagawa University Hospital, Kagawa Prefecture, Japan. Participants answered all the questions. Three respondents were excluded because of psychiatric disorders, and the final number of participants was 91, with a valid response rate of 55.5%. Therefore, the dropout rate was 44.5%. Valid respondents included 52 mothers with a mean age of 32.2 years (SD = 5.0) and 39 fathers with a mean age of 33.9 years (SD = 5.6). Consequently, 38 pairs were identified.

In terms of mothers’ occupation, 13 were office workers (25%), one civil servant (2%), one self-employed (2%), nine housewives (17%), one doctoral student (2%), two part-time workers (4%), six teachers or childcare workers (11%), eight in the medical field (15%), five in hospitality and restaurants (10%), two unemployed (4%), and four others (8%). Seventeen fathers were office workers (44%), six were civil servants (15%), four were self-employed (10%), two were teachers or childcare workers (5%), seven were in the medical field (18%), and three were others (8%).

Of the infants, 28 (50.9%) were boys and 27 (49.1%) were girls, including two sets of twins. Less than half of the parents had one child (n = 24, 45%), 19 had two children (36%), and 10 had three children (19%). In terms of sibling ages, seven were one year old (19%), nine were two years old (24%), three were three years old (8%), seven were four years old (19%), four were five years old (11%), two were six years old (5%), three were seven years old (8%), one was 10 years old (3%), and one was 11 years old (3%).

### 2.2. Procedure

This study was conducted at a university hospital. Individualized paper-based self-administered questionnaires were distributed during the one-month checkup. Self-administered questionnaires were completed on the spot and submitted to a questionnaire collection box placed in the pediatric department at the end of the checkup. If it was difficult to answer the questionnaire immediately, we collected the responses by post mail. We assigned the same research ID to the fathers’ and mothers’ questionnaires, so that the paired data could be reviewed. In Japan, most mothers undergo a one-month checkup to check the health of the baby and mother, and to support the postpartum family. The survey was conducted from 13 May–21 September 2021.

#### Measurements

Postpartum Depression: The Japanese version of the Edinburgh Postnatal Depression Scale (EPDS) was used to assess postpartum depression [27,28]. We used EPDS in this study because it is used worldwide as a scale to assess postpartum depression. The EPDS questionnaire comprises ten statements on a 4-point response scale. Scores range from 0 to 30, with higher scores indicating higher levels of depression. In this study, the Cronbach’s alpha was 0.78, indicating high reliability. Although the EPDS was developed to screen for maternal postpartum depression, it has also been used in several studies to screen for paternal postpartum depression [29,30]. Emerging evidence suggests its effectiveness in screening for paternal postpartum depression [31].

Personality: The Japanese Ten-Item Personality Inventory (TIPI-J) was used [18]. The TIPI-J comprises 10 statements rated on a 7-point response scale. The questionnaire measures the five subscales of the Big Five personality model. We used the TIPI-J because it is extremely simple and has been examined for reliability and validity [18]. Since the participants of this study were parents who were one month postpartum, a large number of items were considered burdensome. The scores for each subscale range from 2 to 14, with higher scores indicating these characteristics to a greater degree. In this study, Cronbach’s alpha was 0.73 for extraversion, 0.03 for cooperativeness, 0.70 for conscientiousness, 0.70 for neuroticism, and 0.49 for openness. We excluded agreeableness and openness from our study because of their low reliability.

Marital satisfaction: The Japanese version of the Quality of Marriage Index (QMI) was used to measure marital satisfaction [32,33]. We used the QMI because it is used globally and is more concise than the other measures of marital satisfaction. The questionnaire instructed participants as follows: “Please answer to what extent these feelings and attitudes apply to your daily life and marital relationship.” The QMI comprised six statements with a 4-point response scale. The scores ranged from 6 to 24, with higher scores indicating higher marital satisfaction. In this study, Cronbach’s alpha was 0.88, indicating high reliability.

Demographic information: The parents’ face items (age, occupation, number of children, age and sex of children) and the mothers’ EPDS data at one month postpartum were obtained from their medical records, eliminating personal information.

### 2.3. Statistical Analysis

We calculated the correlations among all study variables using structural equation modeling (SEM). Statistical analysis was performed using SPSS Statistics 28.0 and IBM SPSS Amos 28.0. Statistical significance was set at *p* < 0.05.

### 2.4. Ethical Consideration

This study was approved by the Ethics Committee of Kagawa University School of Medicine (No. 2021-006) and the Institutional Review Board of the Nagoya University Graduate School of Education and Human Development (No. 24-2339). Written informed consent was obtained from all participants. The survey cooperation request form stated that participation was not mandatory, responses would be kept anonymous, and the results would be statistically processed and handled as group data. This study was funded by the 2021 Kagawa University School of Medical School Priority Project Research.

## 3. Results

### 3.1. Correlations Between Fathers’ and Mothers’ Variables

Table 1 presents the correlations between the study variables. Maternal extraversion was correlated with paternal depression (r = −0.53, *p* < 0.001) and maternal neuroticism was correlated with maternal depression (r = 0.51, *p* < 0.001). Furthermore, paternal extraversion was correlated with paternal postpartum depression (r = −0.34, *p* = 0.033), while maternal postpartum depression was correlated with maternal marital satisfaction (r = −0.29, *p* = 0.037).

We also found that paternal extraversion was correlated with paternal marital satisfaction (r = 0.39, *p* = 0.015), and maternal neuroticism was correlated with maternal marital satisfaction (r = −0.34, *p* = 0.013). Furthermore, significant negative correlations were found between maternal extraversion and neuroticism (r = −0.39, *p* = 0.004).

### 3.2. SEM for Postpartum Depression and Personality

SEM was used to examine the relationship between postpartum depression, personality, and marital satisfaction. A total of 38 pairs (76 participants) were included in the analysis.

We hypothesized that maternal neuroticism would influence postpartum depression among parents. Based on these correlations, we hypothesized that parental extraversion influences paternal postpartum depression.

**Figure 1:**
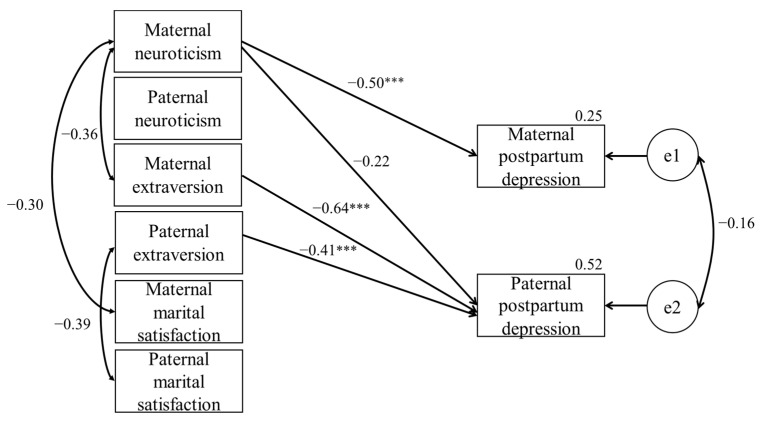
Interdependence model between parents’ personality and postpartum depression. *** *p* < 0.001.

Figure 1 shows the SEM results. The goodness of fit of the model was χ^2^ = 20.639, AIC = 68.639, RMSEA = 0.024, and CFI = 0.986. We observed a significant positive path for maternal neuroticism to maternal postpartum depression (β = 0.50, *p* < 0.001). The path from maternal neuroticism to paternal postpartum depression was not significant (β = −0.22). The analysis also showed a significant negative path for maternal extraversion to paternal postpartum depression (β = −0.64, *p* < 0.001), and for paternal extraversion to paternal postpartum depression (β = −0.41, *p* < 0.001).

We examined whether paired data were similar to unpaired data using a *t*-test. We found no significant differences in age or the measured variables of the EPDS, TIPI-J, and QMI between the paired and unpaired data.

## 4. Discussion

To the best of our knowledge, this is the first study to examine the relationship between parental personality and postpartum depression in Japanese mothers and fathers, using paired data. We examined correlations and analyzed the data using SEM to investigate the association between postpartum depression, personality, and marital relationships in Japanese mothers and fathers at the one-month checkups. Maternal extraversion was moderately associated with paternal depression and maternal neuroticism was moderately associated with maternal depression. Furthermore, paternal extraversion was moderately associated with paternal depression. We found negative weak correlations between marital satisfaction and maternal depression, although no significant paths were found using SEM.

As we found a significantly moderate path from maternal extraversion to paternal depression, this finding indicates that maternal extraversion affects postpartum paternal depression. To our knowledge, this is the first study outside Western countries to show that maternal extraversion is associated with paternal postpartum depression. Previous studies showed that extrovert mothers do not suffer from maternal postpartum depression [34], and extrovert fathers do not suffer from paternal postpartum depression [22]. Our findings indicate that maternal extraversion extends beyond the mother’s intrapersonal personality and affects paternal postpartum depression. Taken together, our findings suggest that when supporting fathers with postpartum depression, it is necessary to consider paternal and maternal personalities.

Moreover, we showed that maternal depression was moderately associated with maternal neuroticism, and paternal depression was moderately associated with paternal extraversion. These results are consistent with those of previous studies showing that maternal neuroticism affects maternal postpartum depression [16,35], and paternal extraversion is associated with paternal postpartum depression [36]. Our findings suggest that personality traits associated with postpartum depression differ between postpartum mothers and fathers. A previous study reported that resilience was the most influential predictor of neuroticism in women and extraversion in men among adolescents [37]. Consistent with previous studies, our findings indicate that the association between postpartum depression and personality traits differs between mothers and fathers. This suggests that a sex-specific approach to interventions for postpartum depression is required [38].

In this study, maternal postpartum depression was weakly associated with maternal marital satisfaction, but no significant pathway was found in SEM. No significant correlation was observed between paternal postpartum depression and marital satisfaction. Previous studies showed that marital relationships for both mothers and fathers are associated with postpartum depression. Nishimura et al. [24] reported a significant positive correlation between paternal satisfaction and depression. Several studies indicated that poor relationships with partners are a risk factor for maternal postpartum depression [25,39]. In addition, maternal marital satisfaction was shown to have a significant mediating effect on postpartum paternal depression [40]. However, as we found no such association in this study, further investigations are required to determine the relationship between marital status and postpartum depression.

We carefully considered the social, historical, and cultural context of parents’ backgrounds when deciding which intervention to use for postpartum depression. Cognitive behavioral therapy (CBT) is a generally recommended intervention for postpartum depression. Previous studies have shown that CBT effectively improves the symptoms and progression of postpartum depression [41,42]. Although numerous studies have demonstrated the effectiveness of CBT, many countries, including Japan, face problems regarding the inadequacy of professionals who can conduct CBT and its maldistribution in urban areas. To overcome these problems, streamlined cognitive–behavioral therapy [43] and Internet cognitive–behavioral therapy (iCBT) [44] have been developed. Furthermore, our findings indicate the need for couple-based support. Previous studies showed that cognitive–behavioral intervention for couples reduces early maternal postpartum depression compared with those who receive it only for mothers [45]. However, healthcare practitioners working with birth trauma emphasize CBT and have described the need to integrate approaches because professionals in the field lack the knowledge and understanding necessary to provide appropriate care [46]. In addition to CBT, we may need to support changes in self-perception due to birth and loss related to birth experiences and autonomy [46]. Our findings are novel and provide significant insights into the health practice of postpartum depression, suggesting that health practitioners should consider each parent’s personality as an effective therapeutic intervention. We believe that healthcare practitioners in the field need to consider the context of parents’ backgrounds, such as personality and marital satisfaction when providing support for postpartum depression.

This study had several limitations. First, we used self-administered questionnaires and included those who responded to all of the questionnaires; therefore, future research should use additional data collection methods, such as interviews with partners. Second, the sample size in this study was small owing to the fixed survey period, and may have been biased because the survey was conducted on couples who had received a one-month checkup at a university hospital during this period. Therefore, our findings should be interpreted with caution in the extent that the result can be generalized. Future studies need to conduct sample estimation and examine broad varieties of parents. Third, we conducted a cross-sectional survey. Therefore, causality should be carefully considered. Fourth, our study did not include confounding factors, such as social support, financial issues, or self-esteem. Fifth, we focused on the couples. It should be noted that we were not able to consider single mother- or single father-infant pairs. Finally, the effects of COVID-19 were not considered in this study as the COVID-19 pandemic, which began in December 2019, occurred during the study year, and has been widely reported to cause maternal and paternal postpartum depression [47,48].

## 5. Conclusions

In this study, we used paired data of mothers and fathers at one month postpartum. Our findings suggest that at one month postpartum, personality traits and marital satisfaction associated with postpartum depression differ between mothers and fathers. These findings are important for developing interventions aimed at preventing postpartum depression in parents. We believe that it is necessary to establish a depression prevention program for mothers and fathers during the perinatal period, considering the characteristics of the perinatal period. Our findings provide important information for developing effective psychological interventions.

## Figures and Tables

**Table 1:** Correlation between mothers’ and fathers’ variables.

	Maternal Postpartum Depression	Maternal Extraversion	Maternal Conscientiousness	Maternal Neuroticism	Maternal Marital Satisfaction	Paternal Postpartum Depression	Paternal Extraversion	Paternal Conscientiousness	Paternal Neuroticism
Maternal extraversion	−0.19								
Maternal conscientiousness	−0.07	0.39 **							
Maternal neuroticism	0.51 ***	−0.39 **	−0.39 **						
Maternal marital satisfaction	−0.29 *	0.12	0.00	−0.34 *					
Paternal postpartum depression	−0.09	−0.53 ***	−0.15	0.17	−0.21				
Paternal extraversion	0.08	−0.09	0.11	−0.20	0.14	−0.34 *			
Paternal conscientiousness	0.13	0.21	0.09	−0.17	0.24	−0.28	0.54 ***		
Paternal neuroticism	0.17	−0.13	−0.47 **	0.27	−0.12	0.22	−0.26	−0.26	
Paternal marital satisfaction	0.14	0.04	0.15	−0.01	0.22	−0.28	0.39 *	0.37 *	−0.16

* *p* < 0.05, ** *p* < 0.01, *** *p* < 0.001.

## Data Availability

These data are not publicly available to protect the confidentiality of the research participants and prevent any potential compromise to their privacy.
